# Acute neuroinflammation provokes intracellular acidification in mouse hippocampus

**DOI:** 10.1186/s12974-016-0747-8

**Published:** 2016-11-03

**Authors:** Anna A. Tyrtyshnaia, Larisa V. Lysenko, Francisco Madamba, Igor V. Manzhulo, Maxim Y. Khotimchenko, Alexander M. Kleschevnikov

**Affiliations:** 1 Department of Neurosciences, University of California San Diego, 9500 Gilman Drive, La Jolla, CA 92093 USA; 2 School of Biomedicine, Far Eastern Federal University, Sukhanova 8, Vladivostok, 690950 Russian Federation; 3 Academy of Biology and Biotechnology of Southern Federal University, 194/1 Stachki Str, Rostov-na-Donu, 344090 Russian Federation

**Keywords:** Acidification, Extracellular pH, Intracellular pH, BCECF, Neuroinflammation, LPS, IL-1β, Iba1, Hippocampus, Mice

## Abstract

**Background:**

Maintaining pH levels within the physiological norm is an important component of brain homeostasis. However, in some pathological or physiological conditions, the capacity of the pH regulatory system could be overpowered by various factors resulting in a transient or permanent alteration in pH levels. Such changes are often observed in pathological conditions associated with neuroinflammation. We hypothesized that neuroinflammation itself is a factor affecting pH levels in neural tissue. To assess this hypothesis, we examined the effects of acute LPS-induced neuroinflammation on intra- and extracellular pH (pHi and pHo) levels in the CA1 region of mouse hippocampus.

**Methods:**

Acute neuroinflammation was induced using two approaches: (1) in vivo by i.p. injections of LPS (5 mg/kg) and (2) in vitro by incubating hippocampal slices of naïve animals in the LPS-containing media (1 μg/mL, 1 h at 35 °C). Standard techniques were used to prepare hippocampal slices. pHi was measured using ratiometric pH-sensitive fluorescent dye BCECF-AM. pHo was assessed using calibrated pH-sensitive micropipettes. The presence of neuroinflammation was verified with immunohistochemistry (IL-1β and Iba1) and ELISA (IL-1β and TNF-α).

**Results:**

A significant reduction of pHi was observed in the slices of the LPS-injected 3-month-old (LPS 7.13 ± 0.03; Sal 7.22 ± 0.03; *p* = 0.043, *r* = 0.43) and 19-month-old (LPS 6.78 ± 0.08; Sal 7.13 ± 0.03; *p* = 0.0001, *r* = 0.32) mice. In contrast, the levels of pHo within the slice, measured in 19-month-old animals, were not affected (LPS 7.27 ± 0.02; Sal 7.26 ± 0.02; *p* = 0.6, *r* = 0.13). A reduction of pHi was also observed in the LPS-treated slices during the interval 3.5–7 h after the LPS exposure (LPS 6.92 ± 0.07; Veh 7.28 ± 0.05; *p* = 0.0001, *r* = 0.46).

**Conclusions:**

Acute LPS-induced neuroinflammation results in a significant intracellular acidification of the CA1 neurons in mouse hippocampus, while the pHo remains largely unchanged. Such changes may represent a specific protective reaction of neural tissue in unfavorable external conditions or be a part of the pathological process.

**Electronic supplementary material:**

The online version of this article (doi:10.1186/s12974-016-0747-8) contains supplementary material, which is available to authorized users.

## Background

Maintaining brain acidity within the physiological norm is an important component of homeostasis in the central nervous system. pH levels affect the conformation of proteins and other biological molecules, thereby regulating the efficiency and functional activity of enzymes, receptors, and ion channels [1–4]. Accordingly, enzymatic reactions, ion transport, and protein and DNA synthesis, as well as the physiological integrity of cells, depend on the pH levels in neural tissue [1, 4–6]. To ensure the functional stability of neural tissue, a powerful multicomponent pH regulatory system has evolved [3, 7]. However, the capacity of this system can become overwhelmed during critical conditions caused by various pathological or even physiological factors resulting in a transient or permanent alternation in brain acidity [7].

Changes in regional pH levels in the brain have been observed in a number of neurological and neurodegenerative disorders. Thus, acidic pH shifts were observed in the basal ganglia and the whole brain in bipolar disorder [8, 9]. Hippocampal pH levels were reduced in mild cognitive impairment (MCI) [10] and even in normal aging [11–13]. Interestingly, all these conditions are characterized by increased neuroinflammation [9, 14] suggesting that neuroinflammation itself could be a factor affecting neural pH levels.

To determine whether neuroinflammation is affecting brain acidity, we measured intra- and extracellular pH levels in mouse hippocampal slices after experimentally induced neuroinflammation. To this end, two approaches were used. First, acute neuroinflammation was induced “in vivo” by a single intraperitoneal injection of bacterial lipopolysaccharides (LPS). Second, neuroinflammation was induced “in vitro” by exposing hippocampal slices of naïve animals to the LPS-containing media. We observed that neuroinflammation was accompanied by a significant reduction of intracellular pH levels in both cases, while the extracellular pH remained largely unchanged. Thus, acute neuroinflammation may cause significant intracellular acidification in a mouse hippocampus. We speculate that such changes represent a protective reaction, specific for neural tissue, which may restrict neuronal activity, thus reducing the impact of pathological factors on the integrity of neural circuits in unfavorable conditions.

## Methods

### Animals

The experiments were performed on male mice bred on C57BL/6JEi × C3SnHeSnJ (B6EiC3) genetic background. The animals were housed two to four per cage with a 12-h light–dark cycle and ad lib access to food and water. To reduce stress, the mice were handled for 5 min once a day during five consecutive days before the experiments. The experiments with in vivo-induced neuroinflammation were performed on 3-month-old (*n* = 12) and 19-month-old (*n* = 10) mice. The experiments with in vitro-induced neuroinflammation were performed on 3-month-old (*n* = 10) animals. All experiments were conducted in accordance with the National Institutes of Health guidelines and with an approved protocol from the University of California San Diego (UCSD) Institutional Animal Care and Use Committee.

### Slice preparation

The animals were anesthetized with isoflurane before decapitation. The brain was quickly removed and immersed for 2 min in ice-cold artificial cerebrospinal fluid (ACSF) containing 119 mM NaCl, 2.5 mM KCl, 2.5 mM CaCl_2_, 1.3 mM MgSO_4_, 1 mM NaH_2_PO_4_, 26 mM NaHCO_3_, and 10 mM glucose, osmolarity 310 mOsm, continuously bubbled with carbogen (95 % O_2_, 5 % CO_2_), at pH 7.4. The hippocampus was extracted and cut in ice-cold ACSF with a vibratome (Leica 1000) into 350-μm-thick slices, which were allowed to recover in oxygenated ACSF at 35 °C for 15 min and then at room temperature for at least 1 h.

### Experimental design

The effects of acute neuroinflammation on neural acidity were measured in mouse hippocampal slices using two approaches.

#### “In vivo”-induced neuroinflammation

The mice were i.p. injected with either LPS (5 mg/kg, 200 μL) or vehicle (0.9 % NaCl, 200 μL). Hippocampal slices were prepared 3 h after the injections, allowed to recover, and loaded with BCECF-AM (5 μM, 30 min at 35 °C). After that, the slices were washed three times in fresh ACSF and stored for 1–6 h in regular ACSF (RT) before the pH measurements.

#### “In vitro”-induced neuroinflammation

Neuroinflammation was induced by incubating hippocampal slices of naïve animals in the LPS-containing media. To this end, the slices were prepared as described, allowed to recover for 50 min, and then transferred for 1 h in small (2 mL) individual vales filled with ACSF containing either LPS (1 μg/mL, *Escherichia coli* 0111:B4, cat. # L4391, Sigma-Aldrich, St. Louis, MO) or vehicle. The solution temperature was constantly monitored with a thermocouple thermometer and kept at 35 °C. To ensure proper oxygenation of the media, the vales were covered with plastic leads and the air/medium surface was constantly overblown by warm fresh carbogen. After this procedure, the slices were washed three times for 5 min in fresh oxygenated warm ACSF and loaded with BCECF-AM (5 μM, 30 min at 35 °C). After the staining, the slices were washed three times and stored in regular ACSF at 25 °C for 0–5 h before the pH measurements.

### Measurements of pH levels

Extracellular pH (pHo) was measured using pH-sensitive micropipettes fabricated as previously described [15]. Briefly, concentric pH-sensitive micropipettes were fabricated from two thin-walled borosilicate glass capillaries of different diameters. The wider capillary had an OD of 2.0 mm and an ID of 1.6 mm (A-M Systems 6185). The inner surface of this capillary was covered with *N*,*N*-dimethyltrimethylsilylamine (Fluka 41720) and filled with proton-selective cocktail (Fluka 95291), which was incorporated into the capillary tip by suction to form a 100- to 200-μm column. The inner micropipette was pulled using a thin-walled glass capillary with an OD of 1.2 mm and an ID of 0.9 mm (A-M Systems 6160) to produce a tip diameter of ~1 μm. The micropipette was then backfilled with a solution of 3 M KCl containing 50 mM K/Na phosphate buffer at pH 7.4. The smaller-diameter micropipette was threaded within the larger barrel and through the column of the ion exchanger, until its end was ~10 μm from the tip of the outer micropipette. The inner barrel was then secured around the opening of the outer barrel using wax. Electrical contact to the KCl solution of the inner pipette was made with a silver–silver chloride junction. Freshly fabricated pH-sensitive micropipettes were calibrated using phosphate buffers with different predetermined pH levels. Because the registered voltage is proportional to the pH levels for such micropipettes [15], two solutions with pH 6.9 and 7.4 were used for the calibration. A representative calibration curve is shown in Additional file 1: Figure S1A. The sensitivity of individual micropipettes (*K*) varied from 56 to 64 mV/pH, with the average value of 60.9 ± 1.0 mV/pH unit.

When measured in slice preparations, the level of pHo varies as a function of distance from the slice surface [16–18]. To measure such pHo profiles, the tip of a calibrated pH-sensitive micropipette was first placed at the “starting” position of 200 μm above the slice, at which position pHo = pHacsf, and then moved down by 20-μm steps, once in 5 s, till the position of 180 μm below the slice surface (Additional file 1: Figure S1B, “Vp,” see also a schematic on Fig. 3a). After recording with the pH-sensitive micropipette, the same procedure was repeated with a regular microelectrode to record the corresponding changes in voltage (Additional file 1: Figure S1B, “Ve”). The pHo levels were computed as pHo = pHacsf - (Vp − Ve)/*K* (Additional file 1: Figure S1C).

Intracellular pH (pHi) was measured using ratiometric pH-sensitive dye BCECF-AM (B1170, Thermo Fisher Scientific, Waltham, MA). To this end, hippocampal slices were loaded with BCECF-AM (5 μM, 30 min at 35 °C), washed in fresh warm ACSF three times for 5 min, and allowed for BCECF-AM processing at RT for additional 0.5–1.5 h before the pH measurements. For the measurements, one of the BCECF-loaded slices was transferred into the submerged recording chamber superfused with ACSF at a constant rate of 2.5 mL/min at 32 °C. After a 10-min stabilization, the slice was excited at 440 and 490 nm using a LED light source (pE-2, CoolLED, UK), and the fluorescence emitted at 535 nm was captured using fluorescent microscope BX-51 (Olympus) equipped with the Rolera-XR (QImaging) digital camera. The images were stored on a computer hard drive and used for offline estimation of pHi using MetaMorph (Molecular Devices, USA).

For quantitative evaluation of pHi, calibration with a modified nigericin method [19] was performed at the end of each experiment. To this end, high-K^+^ (100 mM) solution with nigericin (5 μM) was pressure-applied to the slice surface locally for 10 min through a micropipette (tip diameter ~10 μm) placed ~20 μm above the slice, and the *F*_440_/*F*_490_ ratios were measured in ACSF-pH_1_ (pH = 7.4) and ACSF-pH_2_ (pH = 6.6). Local pHo levels in neurons’ vicinity were measured with a pH-sensitive micropipette, and the dependence of the *F*_440_/*F*_490_ ratio from the pHi was computed. All healthy-looking CA1 pyramidal neurons within the field of view were selected as regions of interest (ROI) using DIC images. The ROI areas varied from 280 to 520 μm^2^. The results were averaged for all ROI in the locus.

### Immunohistochemistry

#### In vivo study

The mice were deeply anesthetized with isoflurane and transcardially perfused for 1 min with 0.9 % NaCl (10 mL) and then for 10 min with 4 % paraformaldehyde in 0.1 M phosphate-buffered saline (PBS), pH 7.4. The brains were removed, kept in the same fixative for 24 h, washed, and then transferred to a 30 % sucrose solution for a day. Thereafter, the samples were transferred to a Neg 50 (Thermo Scientific) medium for another day and then 30-μm sagittal sections were prepared with a cryostat microtome (HM525, Thermo Scientific). Free-floating sagittal sections were pre-incubated in 5 % nonfat milk in PBS and then incubated overnight at 4 °C with rabbit IL-1β (ab9722, Abcam, 1:500) or Iba1 (ab107159, Abcam, 1:500) primary antibody. The sections were then rinsed in PBS (10 min, three changes) and incubated for 2 h at room temperature with peroxidase mouse anti-rabbit IgG (PI-1000, Vector Laboratories, 1:200). After rinsing with PBS (10 min, three changes), the sections were incubated with diaminobenzidine solution (SK-4105, ImmPACT™ DAB Peroxidase Substrate) for 2 min at room temperature. Following further rinsing, the sections were mounted on microscope glass slides and coverslipped with VectaMount Permanent Mounting Medium (H-5000, Vector Laboratories).

Microphotographs were captured and stored as TIFF files. The images were processed and analyzed using ImageJ (NIH, USA). Quantification of IL-1β- and Iba1-immunopositive cells was performed on a one-in-six series of labeled sections. The total number of IL-1β- or Iba1-positive cells in the CA1 region was computed by an operator blinded to the sections’ identity. The number of cells per 1 mm^3^ was computed.

#### In vitro study

To determine whether or not in vitro exposure of hippocampal slices to LPS activated pro-inflammatory signaling, hippocampal slices were prepared as described for pH measurements, allowed for 1-h recovery, and then placed in saline–ACSF or 1 μg/mL LPS–ACSF. After 30 min of drug exposure, the slices were incubated in ACSF for additional 2–7 h and then fixed in 4 % paraformaldehyde in 0.1 M PBS for 12 h. After that, the slices were washed five times for 5 min, and 50-μm-thick sections were made from the central part of the 350-μm slices using a vibratome (Leica 1000). Immunohistochemistry for IL-1β and evaluation of the data were performed as described above.

### Enzyme-linked immunosorbent assay

Enzyme-linked immunosorbent assay (ELISA) was used to quantify the hippocampal levels of IL-1β and TNF-α. In the in vivo experiments, isoflurane-anesthetized mice were decapitated 3 h after the LPS injection. Within 1 min after the decapitation, the hippocampi were extracted, quickly frozen, and stored at −70 °C until use. In the in vitro experiments, the slices collected after the LPS or saline exposure were used. Mouse IL-1β ELISA (ab100705, Abcam) and TNF-α ELISA (ab100747, Abcam) kits were used according to the manufacturer’s recommendations. Neural tissue was homogenized on ice in the extraction buffer recommended by the manufacturer (100 mM Tris, pH 7.4, 150 mM NaCl, 1 mM EGTA, 1 mM EDTA, 1 % Triton X-100, 0.5 % sodium deoxycholate) with 1 mg/mL of protease inhibitor cocktail (cOmplete, Sigma-Aldrich) and 0.01 mg/mL of phosphatase inhibitor cocktail (P5726, Sigma-Aldrich). The protein concentrations were determined using a BCA protein assay kit (Pierce, Rockford, IL). The absorbance at 450 nm was measured with an iMark Microplate Absorbance Reader (Bio-Rad).

### Statistical analysis

Data are shown as mean ± SEM. “*n*” represents the number of images for the pHi measurements, the number of slices for the pHo and IHC measurements, and the number of animals for ELISA. All tests were performed using GraphPad software. All parameters were examined for normality of distributions using the Kolmogorov–Smirnov test. Depending on whether or not the experimental data showed normal distributions, the nonparametric Mann–Whitney rank sum test or one-way analyses of variance (ANOVAs) were used. The differences were considered significant at *p* < 0.05.

## Results

The effects of acute neuroinflammation on neural pH levels were assessed in mouse hippocampal slices using two approaches. First, neuroinflammation was induced in vivo by i.p. injections of LPS, while vehicle-injected littermate mice were used for controls. Second, neuroinflammation was induced in vitro by exposing hippocampal slices of naïve animals to the LPS-containing media. Adjacent slices from the same animal were treated with vehicle (saline) and used for controls. The presence of neuroinflammation was confirmed by immunostaining, for IL-1β and Iba1, and ELISA, for IL-1β and TNF-α.

### In vivo-induced neuroinflammation

**Fig. 1:**
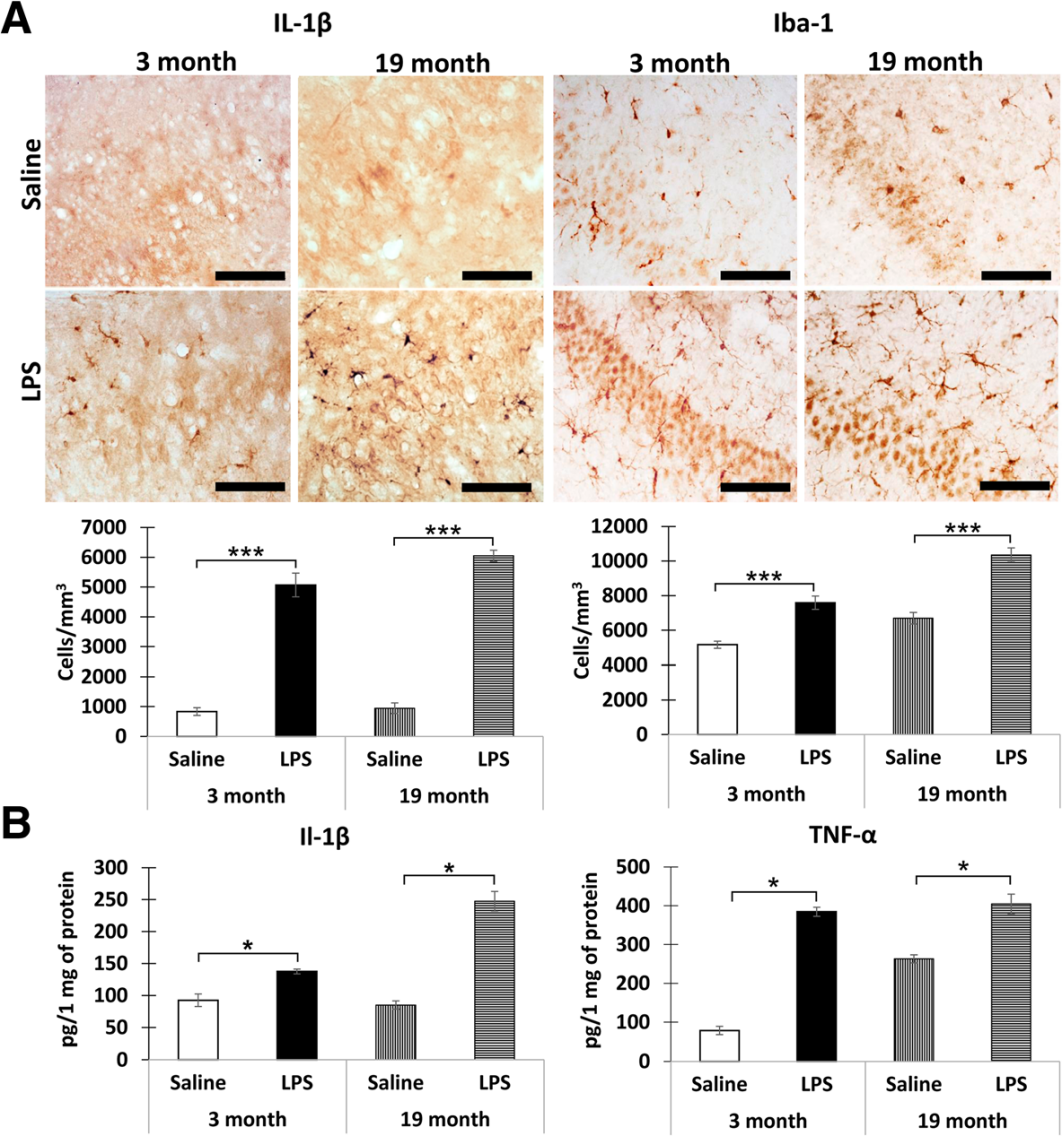
i.p. injection of LPS-induced neuroinflammation in both young and old mice. **a** *Top*: representative examples of IL-1β and Iba1 immunostaining in hippocampal slices from the saline- and LPS-injected mice. The mouse age is indicated *above* the images. *Bottom graphs*: quantification of the cell density. Mean ± SEM, *n* = 10 per group, ***p = 0.0001. **b** Levels of IL-1β and TNF-α measured by ELISA. Mean ± SEM, *n* = 4 per group, *p < 0.03

Intraperitoneal injection of bacterial lipopolysaccharides represents an effective experimental approach to induce neuroinflammation in the mammalian brain. Profound activation of pro-inflammatory markers can usually be observed in a few hours after LPS injections (e.g., [20, 21]). We injected LPS or vehicle in 3- and 19-month-old mice and examined neuroinflammation and neural acidity in hippocampal slices prepared 3 h after the injections. To determine whether the LPS injections induced neuroinflammation, we first used immunohistochemistry to measure the densities of IL-1β- and Iba1-immunopositive neurons in the CA1 region (Fig. 1a). As expected, the densities of both IL-1β- and Iba1-immunopositive cells were significantly increased in the slices from the LPS-injected 3-month-old (IL-1β *F* = 114.4, *p* = 0.0001; Iba1 *F* = 34.5, *p* = 0.0001) and 19-month-old (IL-1β *F* = 424.8, *p* = 0.0001; Iba1 *F* = 53.5, *p* = 0.0001) mice (Fig. 1a). Next, ELISA was used to assess the concentrations of IL-1β and TNF-α. Again, the concentrations of these neuroinflammatory markers were increased in the slices from both 3-month-old (IL-1β *p* = 0.029, *r* = 0.82; TNF-α *p* = 0.029, *r* = 0.82) and 19-month-old (IL-1β *p* = 0.029, *r* = 0.82; TNF-α *p* = 0.029, *r* = 0.82) LPS-injected mice. Thus, in our experimental conditions, i.p. injections of LPS resulted in effective induction of neuroinflammation.

**Fig. 2:**
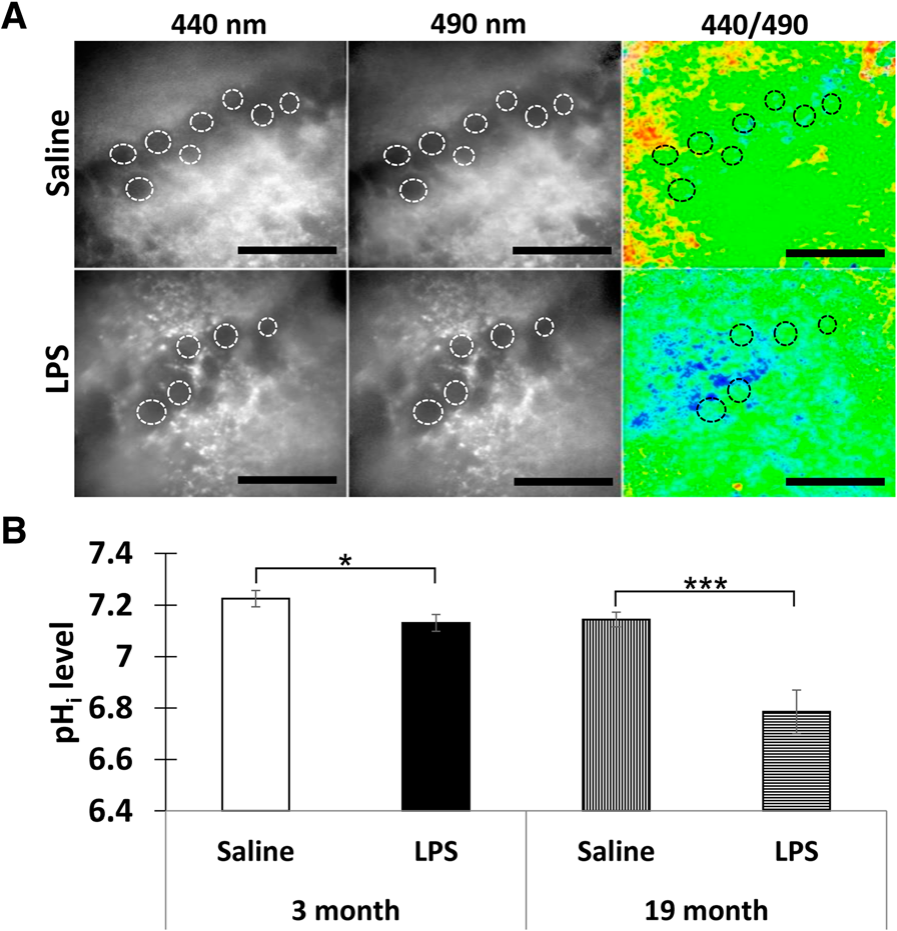
Neuroinflammation induced by i.p. injections of LPS provoked intracellular acidification in the CA1 neurons. **a** Representative examples of ratiometric pHi measurements in the BCECF-AM-loaded slices from 19-month-old mice. The images were taken with the excitation wavelengths 440 and 490 nm, and the *F*_440_/*F*_490_ ratio was assessed. Regions of interest (ROI) used for evaluation of the pHi levels are shown by *circles. Bar* = 100 μm. **b** Quantification of the pHi levels. Mean ± SEM. *n* = 41 (3-month saline), 60 (3-month LPS), 10 (19-month saline), and 12 (19-month LPS); *p = 0.04, ***p = 0.0001

To assess the effects of neuroinflammation on brain acidity, we first measured pHi in the CA1 neurons. To this end, freshly prepared hippocampal slices were loaded with BCECF-AM and the pHi was measured between 3.5 and 7 h after the LPS injections. As can be seen from the representative images (Fig. 2a), the *F*_440_/*F*_490_ ratio was considerably reduced in the slices from the LPS-injected mice suggesting an acidification of the intracellular compartments. These changes were assessed quantitatively after calibrating the *F*_440_/*F*_490_ ratio using a modified nigericin method. The averaged data between 3.5 and 7 h after LPS injections showed a significant reduction of pHi in the LPS vs. saline group in both 3-month-old (*F* = 4.64, *p* = 0.04) and 19-month-old (*F* = 21.19, *p* = 0.0001) mice (Fig. 2b). Thus, the induced neuroinflammation was accompanied by significant intracellular acidification in the CA1 region.

**Fig. 3:**
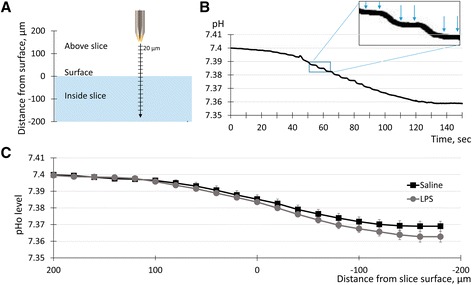
Neuroinflammation induced by i.p. injections of LPS had no effect on extracellular acidity in the CA1 region. **a** Schema of the experiment. A pH-sensitive micropipette was sequentially positioned at distances varying from +200 to −180 μm from the slice surface, in steps of 20 μm, at which pHo levels were measured. **b** Time course of pHo changes during a representative experiment. The micropipette was moved to a new position every 5 s. pHo levels changed during the movements but were stable between the movements. *Insert*: part of the curve at a higher magnification. At each electrode position, pHo levels were averaged for the stable period (*between the arrows in the insert*). **c** Averaged data for the pHo profiles in slices from the saline- and LPS-injected mice. No statistical difference was observed between the saline and LPS groups. LPS: *n* = 10; saline: *n* = 10

Changes in pHi could be generated either by an accumulation of lactate and other acidic products of metabolism or by transmembrane ionic fluxes via ionic transporters and channels. If changes in transmembrane fluxes are involved, it is expected that pHi and pHo would change in the opposite direction and, therefore, intracellular acidification would be accompanied by an extracellular alkalization. On the contrary, if the major cause of pHi changes is metabolic acidosis, pHi and pHo would both show an acidic shift. To assess which of these mechanisms were likely to contribute to the intracellular acidification during neuroinflammation, we measured pHo in the CA1 region using pH-sensitive micropipettes. These measurements were performed on 19-month-old mice, which had showed greater pHi changes. Previously, it was observed that pHo changes gradually as a function of the distance from the slice surface [17, 18]. To measure pHo profiles, we placed the tip of the pH-sensitive micropipette 200 μm above the slice surface and then moved the tip downwards in 20-μm increments until it was located 180 μm below the slice surface (Fig. 3a). A representative time course of the pHo changes during an experiment is shown in Fig. 3b. pHo levels showed step-like changes corresponding to the incremental micropipette movements with relative stability between the movements (Fig. 3b insert, between the arrows). The averaged values for such stable periods were taken as the pHo measure at the corresponding distances from the slice surface. To quantify the effects of neuroinflammation, pHo profiles were averaged for the LPS and vehicle groups (Fig. 3c). For all distances, there was no significant difference between the groups. We concluded that acute neuroinflammation had no effect on pHo in the CA1 region of mouse hippocampus.

Thus, acute neuroinflammation, evoked by i.p. injections of LPS, resulted in significant intracellular acidification without changing the extracellular pH in mouse hippocampus.

### In vitro-induced neuroinflammation

**Fig. 4:**
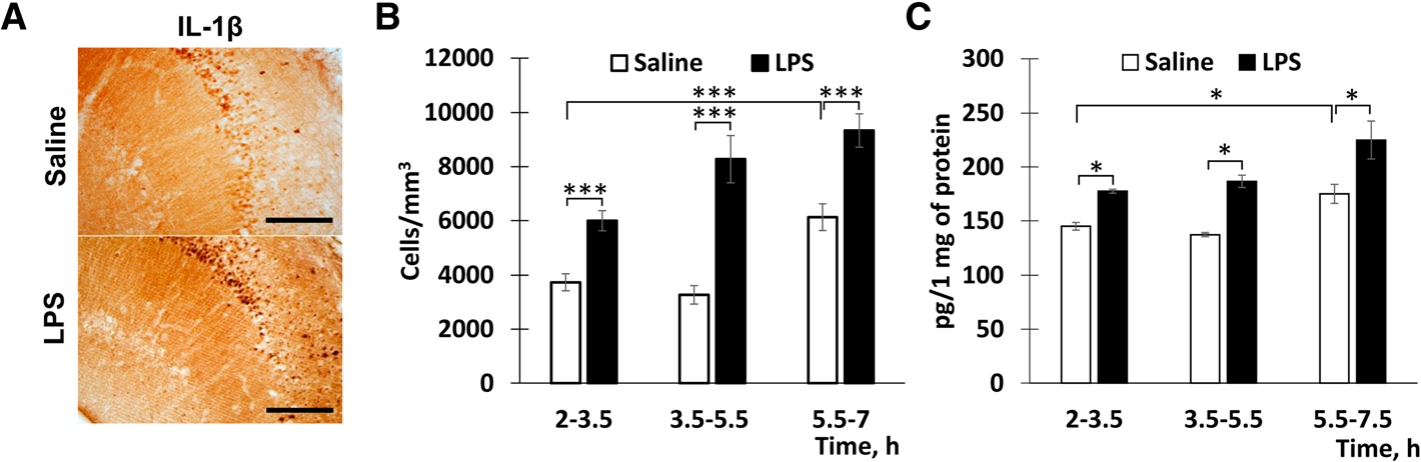
In vitro exposure of hippocampal slices to the LPS-containing media resulted in profound neuroinflammation. **a** Representative images of IL-1β immunostaining in slices treated in vitro with the LPS- (1 μg/mL) or vehicle-containing media. *Bar* = 100 μm. **b** Quantification of the data for IL-1β immunostaining. The mean densities of IL-1β-positive cells at different time intervals after the LPS or vehicle exposure. Mean ± SEM, *n* = 10, *** *p* = 0.0001. **c** Levels of IL-1β measured by ELISA. Mean ± SEM, *n* = 4 per group, *p < 0.03

Next, the effects of in vitro-induced neuroinflammation on pH levels were examined in 3-month-old mice. A notable advantage of in vitro approaches is the possibility for a side-by-side comparison of the slices from the same animal. Such an experimental arrangement allows us to reduce or eliminate the variability caused by individual differences in the animal health, sensitivity to exogenous compounds, etc. To test the feasibility of an in vitro approach to induce neuroinflammation, we first measured the density of IL-1β-positive cells in the CA1 region of slices treated with either LPS (1 μg/mL) or vehicle (Fig. 4a, b). In the LPS-treated slices, the density of IL-1β-positive cells showed a trend for an increase soon after the LPS exposure (2–3.5 h) and increased significantly during the later periods (3.5–5.5 and 5.5–7 h) after the LPS exposure (Fig. 4b). Such changes in the density of IL-1β-positive cells correspond well with the known time course of neuroinflammatory markers during acute neuroinflammation [20, 21]. In the vehicle-treated slices, the density of IL-1β cells was not altered until at least 5.5 h after the treatment and increased only slightly during the last period, 5.5–7 h after the treatment (Fig. 4b). Such an increase could reflect a non-specific inflammation caused by tissue damage during the slice preparation. In order to further verify the induction of neuroinflammation, ELISA was used to measure the levels of IL-1β. Again, the levels of this neuroinflammatory marker were significantly increased in the LPS-treated slices compared to the vehicle-treated slices (Fig. 4c). Thus, in vitro incubation in the LPS-containing media effectively activated neuroinflammatory signaling in mouse hippocampal slices.

**Fig. 5:**
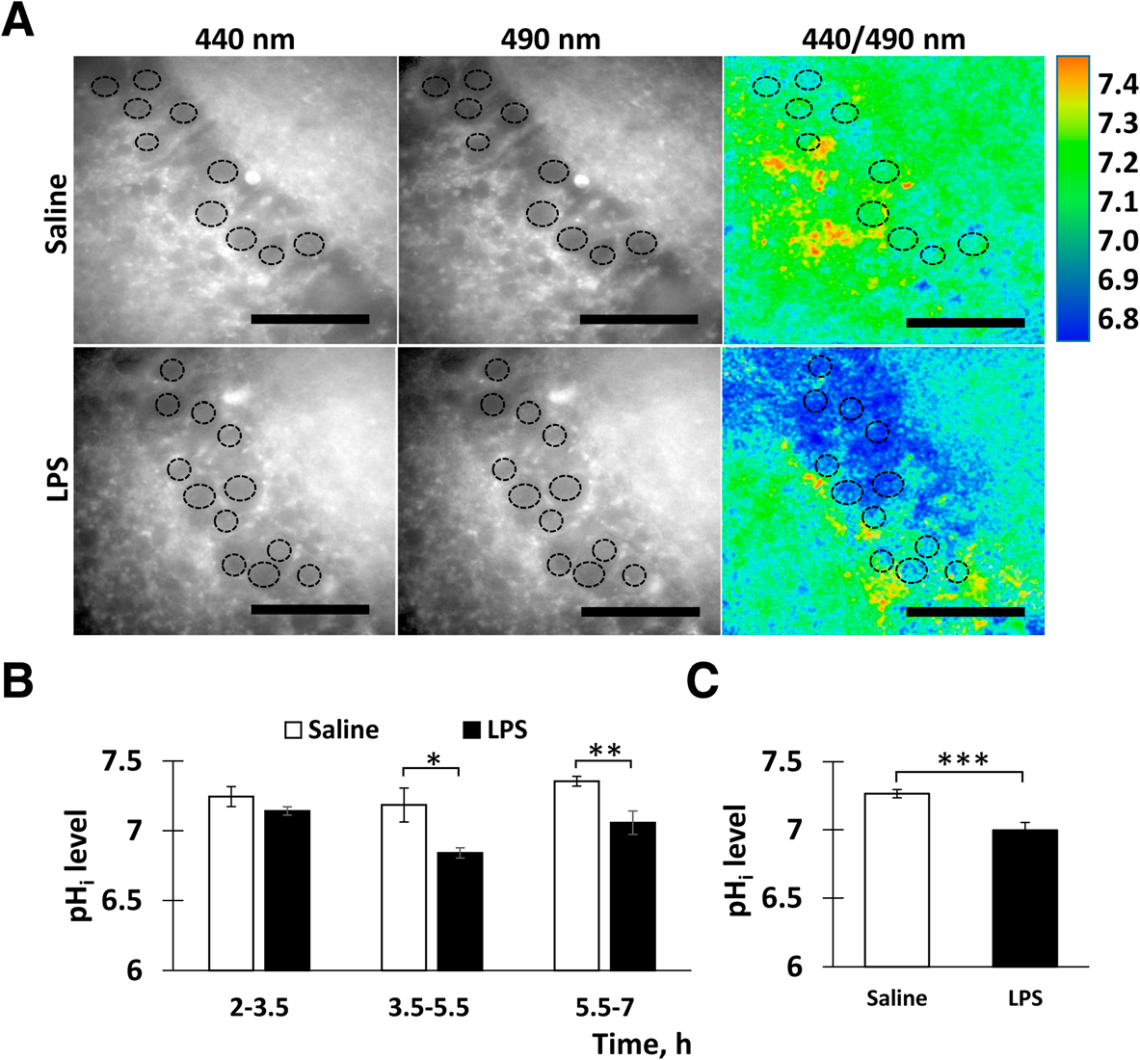
Neuroinflammation induced by in vitro exposure of hippocampal slices to LPS provoked intracellular acidification in CA1. **a** Representative images of the BCECF-AM-loaded slices treated with saline or LPS taken with the excitation wavelengths 440 and 490 nm and the *F*_440_/*F*_490_ ratio. ROI used for the pHi measurements are marked by *circles. Bar* = 100 μm. **b** Quantification of the data. Averaged pHi levels in the saline- and LPS-treated slices at different post-treatment intervals. Mean ± SEM; saline: *n* = 37 (2–3.5 h), 14 (3.5–5.5 h), and 21 (5.5–7 h); LPS: *n* = 18 (2–3.5 h), 22 (3.5–5.5 h), and 14 (5.5–7 h); *p = 0.03, **p = 0.001. **c** Quantification of the data. The data averaged across the entire visible part of the CA1 Str.Pyr. for the period of 3.5–7 h. Mean ± SEM; saline: *n* = 72 (saline) and 54 (LPS); ***p = 0.0001

To measure the effects of the in vitro-induced neuroinflammation on pHi levels, adjacent hippocampal slices were exposed to either LPS- or vehicle-containing media and then loaded with BCECF-AM. After an additional incubation period of 1–6 h, the slices were transferred to the recording chamber for the pHi measurements. Each slice was used for a measurement at a single time point after the treatment. Figure 5a illustrates the pHi measurements in the slices incubated 4 h after the LPS or vehicle exposure. As can be seen from these examples, pHi levels were significantly reduced in the LPS vs. vehicle-treated slices (Fig. 5a). Quantification of the data showed that pHi was stable during the initial period (2–3.5 h) and then significantly reduced (Fig. 5b). Importantly, the most significant pHi reduction was observed for the period 3.5–5.5 h after the LPS exposure, during which the neuroinflammation was observed only in the LPS but not the vehicle-treated slices (see Fig. 4b). Averaged for the total 3.5–7-h period, pHi levels were in Veh 7.29 ± 0.05; in LPS 6.92 ± 0.07, thus showing a difference of ~0.36 pH units (*p* = 0.0001, *r* = 0.46). These results show that neuroinflammation reduces pHi levels not only after in vivo injections of LPS but also after exposing the slices to LPS in vitro.

## Discussion

Here, we examined the effects of acute LPS-induced neuroinflammation on extra- and intracellular pH in the CA1 neurons of mouse hippocampus. We observed that i.p. injections of LPS, which effectively activated pro-inflammatory signaling, provoked significant intracellular acidification in the CA1 neurons. This change was observed in the absence of notable alterations in the extracellular pH levels. Furthermore, induction of neuroinflammation by direct exposure of hippocampal slices from the untreated animals to the LPS-containing media also resulted in intracellular acidification. We suggest that such changes may reflect a protective reaction of neural tissue in harsh conditions or represent a part of the pathological process associated with neuroinflammation.

Tertiary structure and, therefore, functional efficiency of many proteins and other biological molecules depends on the pH of the surrounding tissue. Thus, changes in pH could modify the efficiency of enzymes [22], receptors [5, 23–26], ionic pumps [4, 27, 28], and ionic channels [29–31]. Many pH-sensitive molecules are membrane-bound and involved in the regulation of cellular excitability. As a result, pH changes may reduce or increase neuronal activity in a tissue-specific manner [4, 32]. In the hippocampus, acidification of neural tissue mostly reduces neural excitability [33, 34]. For example, inhibition of monoamine oxidase-A (MAO-A) by the selective inhibitor moclobemide caused a reduction in pHi by 0.1–0.3 pH units and a suppression of both spontaneous and evoked activity of the CA3 neurons in rodent hippocampus [35]. Similar effects were observed following the inhibition of transmembrane acid extrusion by amiloride, an inhibitor of Na^+^/H^+^ exchanger [36], or administration of sulthiame, an inhibitor of carbonic anhydrase [37]. In contrast, a stronger (0.8–1.0 pH) intracellular acidification in crayfish slow flexor muscle fibers increased cellular excitability and provoked all-or-none calcium spikes [32, 38]. In these studies, however, pHo levels were not assessed and, therefore, it remains uncertain whether the pHi changes alone were responsible for the alteration of cellular excitability. Because downregulation of neuronal excitability in harsh conditions improves cell survival, reduction in pHi may play a protective role in neurodegenerative and other disorders.

Local acidosis is a characteristic feature of an inflammatory process on the periphery. Acidic pH shifts were observed in tumors [39], as well as in traumatic and ischemic loci that are characterized by acute or chronic inflammation [40–42]. In the brain, changes in acidity were observed in a number of neurological conditions associated with neuroinflammation, in many of which a reduction of pH has been observed. For example, intracellular pH levels were reduced in the frontal lobes, basal ganglia, and whole brain of patients with bipolar disorder [8, 43]. A reduction of pH was also observed in human hippocampus during normal aging [11–13]. Likewise, intracellular pH was reduced in patients with mild cognitive impairment (MCI) [10]. Interestingly, there was no alteration in the pH levels in patients with Alzheimer’s disease (AD) [10]. Moreover, the AD patients showed an increased intracellular pH in the left hippocampus when compared with the MCI patients [10]. Because all these conditions are characterized by neuroinflammation [9, 44–46], it is plausible to suggest that neuroinflammation may contribute to the pH changes in neurodegenerative disorders. At present, the effects of neuroinflammation on brain acidity have not been yet fully characterized.

We assessed the effects of LPS-induced neuroinflammation on neural pH levels. In our experiments, control pHi and pHo levels were about 7.1 and 7.3, respectively. These values are consistent with the measurements observed in other studies [3, 16, 47, 48] indicating that the slices were in good health in our experimental conditions. Acute neuroinflammation, induced by either i.p. injections or direct administration of LPS in the supporting media, resulted in a reduction of pHi by about 0.1–0.3 pH units leaving pHo unchanged in both cases. It must be noted that because the pH measurements were performed at temperatures lower (~32 °C) than the physiological norm, neuroinflammation-induced pH changes in alive animals may differ in magnitude from those observed in our study.

A reduction of pHi without a notable change in pHo argues against a purely metabolic cause of the pH changes during neuroinflammation and suggests the involvement of active regulatory processes. Indeed, during the pathological accumulation of acidic metabolic by-products, such as lactate and CO_2_, acidic changes should be registered both intra- and extracellularly. For example, nearly equal changes in extra- and intracellular pH have been observed in hippocampal slices during anoxia [49].

Investigation of the mechanisms involved in the pH reduction during neuroinflammation was not a subject of this study. However, some mechanisms responsible for these changes could be suggested. The most straightforward mechanism may rely on reduced function of mitochondria [50]. Indeed, release of reactive oxygen and nitrogen species (ROS and RNS) by activated microglia induces oxidative and nitrosative stress leading to mitochondrial impairment [51, 52]. Moreover, LPS-induced neuroinflammation results in a transient increase in respiratory capacity and ATP production [53], which may also exacerbate the metabolic acidosis. Other mechanisms by which neuroinflammation may affect intracellular pH may include modulation of acid loaders and/or acid extruders. One hypothetical mechanism may rely on activation of neuronal plasma membrane Ca^2+^-ATPase (PMCA), a ubiquitous transporter exchanging intracellular Ca^2+^ for extracellular H^+^. In this scenario, upregulation of IL-1β increases calcium flux through the NMDA receptors [54], thus increasing internal Ca^+2^, while activated PMCA exchanges the internal Ca^+2^ on external H^+^ leading to intracellular acidification. In the extracellular space, a reduction in proton concentration could be compensated by an H^+^ efflux from the activated microglia via a Na^+^/H^+^ exchanger isoform 1 (NHE1)-dependent mechanism [55, 56]. As a result of this compensation, pHo remains unchanged.

The reduction of intracellular pH during neuroinflammation may either lead to neuronal death, a part of the pathological process, or represent a protective reaction reducing neuronal activity and the inflammation-induced damage to the neural system. Indeed, a significant (pH ~0.4) intracellular acidification has been observed as an early event in both death receptor-mediated and mitochondria-dependent types of apoptosis (reviewed by [57]). Such changes may lead to activation of caspase-3, which reaches a maximum efficiency at pH 6.6–6.8 [58] and activation of deoxyribonuclease II [59]. Such reduction in intracellular pH may also provoke caspase-independent neuronal death [60]. On the other hand, the most notable immediate physiological effect of a moderate pHi reduction is a suppression of neuronal activity [33, 34]. This suggests that a moderate acidification may play a protective role in some conditions. For example, NMDA-induced superoxide production and neuronal death were prevented by intracellular acidification by as little as 0.2 pH units [61]. A protective role of lactate [62] could also be linked to moderate intracellular acidification [63]. Interestingly, a reduction of intracellular pH was evoked by several pharmacological compounds, such as antipsychotics (haloperidol, clozapine, ziprasidone), antidepressants (amitriptyline, doxepin, citalopram), anticonvulsants (tiagabine), and other neuroprotective drugs [35, 37, 47]. It was suggested that the intracellular pH reduction may represent one of the mechanisms responsible for the neuroprotective properties of these compounds [47]. It is therefore feasible that inflammation-induced intracellular acidification may represent a specific protective response of neural tissue diminishing cellular activity and over-activation of neurons in harsh conditions.

## Conclusions

Here, we observed that acute LPS-induced neuroinflammation is accompanied by a moderate intracellular acidification of the CA1 neurons in mouse hippocampus. Presumably, such changes may represent a specific protective reaction of neural tissue during neuroinflammation allowing neurons to survive in unfavorable conditions. Alternatively, the reduction of intracellular pH may represent a part of the pathological process.

## Additional file

Additional file 1: Figure S1. Demonstration of pHo measurements. A. A calibration curve built using two phosphate buffers (pH 6.9 and 7.4). Such calibration curves were built for each of the pH-sensitive micropipettes. B. Recordings of voltage profiles with a pH-sensitive (Vp) and a regular (Ve) micropipette. The pH-sensitive micropipette was first placed at the starting position 200 μm above the slice surface and then moved down by 20-μm steps to the position −180 μm below the slice surface. The same procedure was repeated with the regular microelectrode (Ve). The difference between the Vp and Ve values was used to compute pHo at each distance from the slice surface, thus generating a pHo profile. C. Profile of pHo. The values were computed as pHo = (Vp − Ve)/*K*.

## Abbreviations

ACSF: Artificial cerebrospinal fluid
AD: Alzheimer’s disease
LPS: Lipopolysaccharides
MCI: Mild cognitive impairment
NHE1: Na^+^/H^+^ exchanger isoform 1
PBS: Phosphate-buffered saline
pHi: Intracellular pH
pHo: Extracellular pH
RNS: Reactive nitrogen species
ROS: Reactive oxygen species
