# Supplementary figures and images for: Acute neuroinflammation provokes intracellular acidification in mouse hippocampus
Source: J Neuroinflammation. 2016 Nov 3;13:283. doi: 10.1186/s12974-016-0747-8 (PMC5094044; doi:10.1186/s12974-016-0747-8)

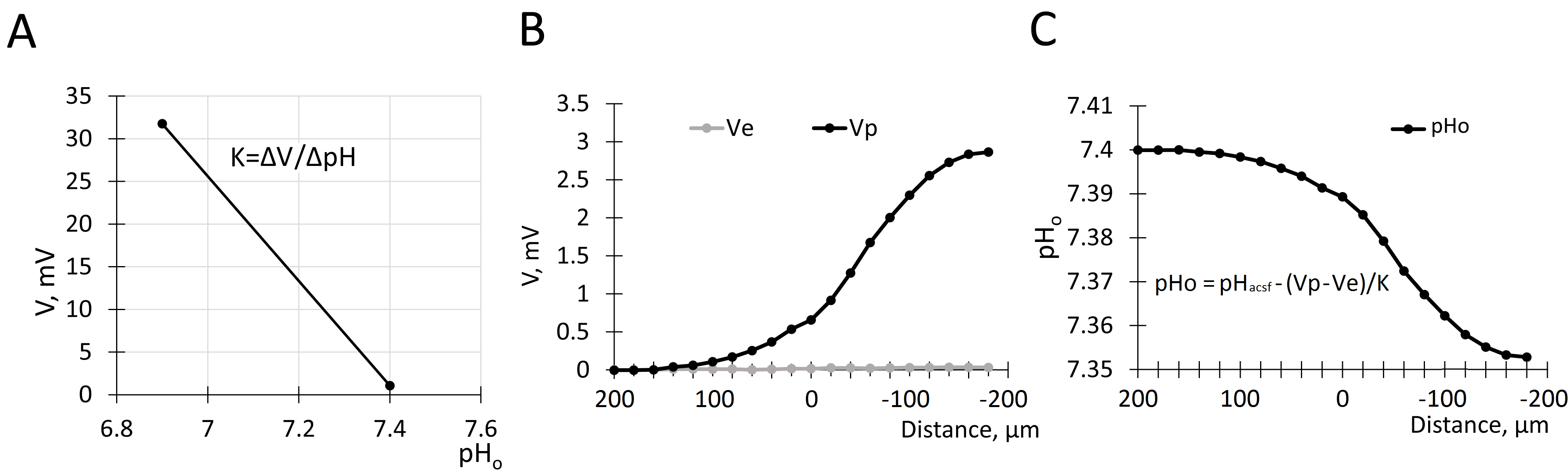

Supplement: Additional file 1: Figure S1. — Demonstration of pHo measurements. A. A calibration curve built using two phosphate buffers (pH 6.9 and 7.4). Such calibration curves were built for each of the pH-sensitive micropipettes. B. Recordings of voltage profiles with a pH-sensitive (Vp) and a regular (Ve) micropipette. The pH-sensitive micropipette was first placed at the starting position 200 μm above the slice surface and then moved down by 20-μm steps to the position −180 μm below the slice surface. The same procedure was repeated with the regular microelectrode (Ve). The difference between the Vp and Ve values was used to compute pHo at each distance from the slice surface, thus generating a pHo profile. C. Profile of pHo. The values were computed as pHo = (Vp − Ve)/K. [file 12974_2016_747_MOESM1_ESM.tif]
